# Inter-Model Comparison of the Landscape Determinants of Vector-Borne Disease: Implications for Epidemiological and Entomological Risk Modeling

**DOI:** 10.1371/journal.pone.0103163

**Published:** 2014-07-29

**Authors:** Alyson Lorenz, Radhika Dhingra, Howard H. Chang, Donal Bisanzio, Yang Liu, Justin V. Remais

**Affiliations:** 1 Department of Environmental Health, Rollins School of Public Health, Emory University, Atlanta, Georgia, United States of America; 2 Department of Biostatistics and Bioinformatics, Rollins School of Public Health, Emory University, Atlanta, Georgia, United States of America; 3 Department of Environmental Sciences, Emory University, Atlanta, Georgia, United States of America; 4 Program in Population Biology, Ecology and Evolution, Graduate Division of Biological and Biomedical Sciences, Emory University, Atlanta, Georgia, United States of America; The Pirbright Institute, United Kingdom

## Abstract

Extrapolating landscape regression models for use in assessing vector-borne disease risk and other applications requires thoughtful evaluation of fundamental model choice issues. To examine implications of such choices, an analysis was conducted to explore the extent to which disparate landscape models agree in their epidemiological and entomological risk predictions when extrapolated to new regions. Agreement between six literature-drawn landscape models was examined by comparing predicted county-level distributions of either Lyme disease or *Ixodes scapularis* vector using Spearman ranked correlation. AUC analyses and multinomial logistic regression were used to assess the ability of these extrapolated landscape models to predict observed national data. Three models based on measures of vegetation, habitat patch characteristics, and herbaceous landcover emerged as effective predictors of observed disease and vector distribution. An ensemble model containing these three models improved precision and predictive ability over individual models. *A priori* assessment of qualitative model characteristics effectively identified models that subsequently emerged as better predictors in quantitative analysis. Both a methodology for quantitative model comparison and a checklist for qualitative assessment of candidate models for extrapolation are provided; both tools aim to improve collaboration between those producing models and those interested in applying them to new areas and research questions.

## Introduction

A range of human and ecological risk assessment activities involve applying quantitative knowledge—such as a model and its parameters drawn from previous work—to a new research question or analytical problem (conceptual extrapolation), or to a new geographic region or time period (spatial or temporal extrapolation). The resulting application outside the conceptual, spatial or temporal domain of the original analysis is an extrapolation, in one or more dimensions, that adds uncertainty to the resulting risk estimates [1], [2]. Examples of quantitative information routinely drawn from previous work include mathematical models and their parameters, dose-response functions, and thresholds and other parameter estimates [1], [3]. Common applications of such information include health impact assessments [4], [5], ecological risk assessments [6], [7], and risk mapping of disease vectors [8], [9].

With growing interest in quantifying shifts in the spatial distribution of hazards, such as disease vector populations, in response to environmental change, models and their associated parameters that describe the environmental dependence of hazards are needed [10]–[13]. In many cases, these are drawn from previous work unrelated to environmental change, and this is especially true for relationships between landscape characteristics and infectious disease vectors, hosts, and reservoirs. Ecological landscape regression models and their parameters are of increasing relevance to, and are increasingly used by, public health risk assessors who seek a quantitative understanding of the potential for changes in the distribution, timing, and intensity of vector-borne diseases under future environmental conditions [14]–[16]. Predictions of future distributions of vectors, for instance, can aid in identifying areas to target for future funding and intervention [17].

Applying models, and landscape models in particular, to describe the distribution of important vector and reservoir species to regions, times, and climates that fall outside the ranges in which the original models were fit raises a unique set of model extrapolation issues surrounding the choice of model for extrapolation. When sufficient computational resources and data are available, model choice may be made by quantitative comparison of multiple candidate models' outputs against field conditions observed outside the domain of the original model fitting. Such comparisons from areas such as climate science, environmental science, physiology, and economics have revealed significant variability in model predictions when modeling methods, resolution, predictor variables and other aspects differ [18]–[21]. Where it is not possible for all candidate models to be recreated, extrapolated, and compared, subjective examination of model characteristics can guide model choice. Here, we describe and demonstrate the relevance of these characteristics by extrapolating multiple existing landscape models (Table 1) of *Ixodes scapularis*, the primary tick vector of Lyme disease in the Eastern U.S. We examine the extrapolation issues summarized in Text S1, and provide a checklist (Table 2) for qualitative assessment of candidate models for extrapolation. This tool, valuable both to model consumers and to model producers, is intended to improve the interaction between those building generalizable models and those with interest in applying them to new areas and research questions.

**Table 1:** Habitat models included in inter-model comparison.

Model Name and Reference	Location	Outcome	Predictors	Model Type	Model*	Parameter Estimates
Tick Patch (Brownstein et al., 2005)	Southern Connecticut (12 towns)	Tick density	Patch size, Patch isolation	Poisson	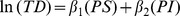	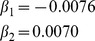
Lyme Patch (Brownstein et al., 2005)	Connecticut (all counties)	Human incidence	Patch size, Patch isolation	Poisson	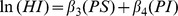	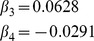
Development (Glass et al., 1995)	Baltimore County, MD	Odds of Lyme disease	Extent of development	Binomial∧	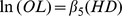	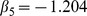
Coniferous (Glass et al., 1995)	Baltimore County, MD	Odds of Lyme disease	Soil that supports coniferous habitat	Binomial∧	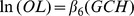	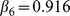
Herbaceous (Glass et al., 1995)	Baltimore County, MD	Odds of Lyme disease	Soil that supports herbaceous habitat	Binomial∧	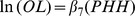	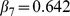
NDVI (Ogden et al., 2006)	Quebec, Canada	Number of ticks submitted	Average NDVI, Population	Negative binomial	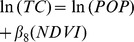	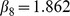

*Intercepts with no parameter estimate provided were not included; TD = tick density; PS = patch size; PI = patch isolation;

∧ Model employed a logistic link function.

HI = human incidence; OL = odds of Lyme disease; HD = highly developed land; GCH = soil classified as fair-good coniferous-supporting habitat; PHH = soil classified as poor-fair herbaceous-supporting habitat; TC = tick count; POP = population; NDVI = normalized difference vegetation index.

**Table 2:** Inter-model comparison considerations and questions applied to Lyme disease incidence and tick abundance/presence models and supporting references.

Criteria	Questions applied to inter-model comparison of Lyme disease/Tick presence models	NDVI Model	Herbaceous Model	Coniferous Model	Development Model	Tick Patch Model	Lyme Patch Model
***CONCEPTUAL EXTRAPOLATION***							
*General characteristics of original analysis*							
Modeling technique	Is the model type appropriate/acceptable to the present research question? Is the model parsimonious? Is the choice of model supported by previous findings or theories?	**Y**	**Y**	**Y**	**Y**	**Y**	**Y**
Suitable species under study	Does the model describe the same or a closely related species?	**Y**	**Y**	**Y**	**Y**	**Y**	**Y**
Model evaluation method	In the original analysis, was data reserved for validation?	N	**Y**	**Y**	**Y**	N	N
Model evaluation method	If so, was reserved data available in different locations, time periods, etc?	N	**Y**	**Y**	**Y**	N	N
*Predictor and outcome variables*							
Direct predictors	Are the model's predictor variables directly involved, in the new analysis, in some biological process of the organism of interest?	N	N	N	N	N	N
Indirect predictor	If not a direct predictor, is the model's indirect variable associated with a variable that directly impacts the biological processes of the organism of interest, under the new research question?	**Y**	**Y**	**Y**	**Y**	**Y**	**Y**
Spatial correlation	Is spatial correlation between the predictor an outcome variable considered?	**Y**	N	N	N	**Y**	**Y**
Data type & appropriate categorization	Is the data type (continuous, categorical, nominal, ordinal etc.) appropriate for the candidate model and the proposed new question?	**Y**	N	N	N	**Y**	**Y**
Relevance of outcome	Is the model outcome variable able to answer the new question?	**Y**	N	N	N	**Y**	N
*Scale*							
Grain	Is the geographic scale of the model appropriate to the question asked in the original analysis?	**Y**	N	N	N	N	N
Grain	Is the grain appropriate to the question being asked in the new analysis?	**Y**	N	N	N	N	N
Time	Are the time scales and time periods of the original analysis appropriate to the new research question?	**Y**	**Y**	**Y**	**Y**	**Y**	**Y**
Methods description	Is the methods description of the original analysis complete? Is it reproducible?	**Y**	**Y**	**Y**	N	**Y**	**Y**
Modeling tools	Are the programs and versions used in the original analysis available for application to the new research area or question?	**Y**	**Y**	**Y**	**Y**	N	N
*Data*							
Data quality	What was the quality of data used in the original analysis? Was the original model fit to high quality data of sufficient quantity?	**Y**	**Y**	**Y**	**Y**	**Y**	**Y**
Data quality	What is the quality of data available for the present analysis?	**Y**	**Y**	**Y**	**Y**	**Y**	**Y**
Availability of data for time period under study	For the present question, is the correct data available in an appropriate data type format?	**Y**	**Y**	**Y**	**Y**	**Y**	**Y**
Availability of data for time period under study	For extrapolation, is data available at a grain similar to the model's original analysis?	**Y**	**Y**	**Y**	**Y**	**Y**	**Y**
***SPATIAL EXTRAPOLATION***							
*General characteristics of original analysis*							
Location	Is the geographic location of the original analysis at the given time period similar to or the same as the location of present analysis?	N	N	N	N	N	N
*Spatial aspects of model variables*							
Presence of variables in new areas	Do the model's variables have values in the new location?	**Y**	**Y**	**Y**	N	N	N
Direct predictors	In the new location, are the model's predictor variables directly involved in some biological process of the organism of interest?	N	N	N	N	N	N
Indirect predictors	If not, is the model's indirect variable strongly associated with a variable that in the new location impacts the biological processes of the organism of interest?	**Y**	**Y**	**Y**	**Y**	**Y**	**Y**
Numerical range of variables in new areas	Is the numerical range of variables in new areas within the range in which the model was fit?	N	N/A	N/A	N/A	N	N
Numerical range of variables in new areas	Is there sufficient variety in values of the variables in the new location to create useful variation in the outcome variable?	N	N	N	N	N	N
Relevant categorization	Is the categorization of the available data relevant and sufficiently descriptive in the new location?	N/A	Y	Y	N	N/A	N/A
Stationarity	Does the model demonstrate stationarity?	ND	ND	ND	ND	ND	ND
Spatial correlation	Are potential changes in spatial correlation accounted for?	**Y**	**Y**	**Y**	**Y**	**Y**	**Y**
*Scale*							
Extent	Does the model cover a sufficient geographic extent in comparison to the extent of the extrapolation?	N	N	N	N	N	N
*Data*							
Availability of data across extrapolation zone	Is the correct data available for the new location in an appropriate data type format?	**Y**	**Y**	**Y**	**Y**	**Y**	**Y**
Availability of data across extrapolation zone	For extrapolation, is data available at a grain similar to the model's original analysis?	**Y**	N	N	N	N	N
Quality of data across extrapolation zone	What is the quality of data available for the present analysis?	**Y**	**Y**	**Y**	**Y**	**Y**	**Y**

Y =  Yes, N =  No, ND =  Not determined, N/A =  Not applicable.

## Materials and Methods

### *Ixodes scapularis* Models

The large number of geographically limited landscape models for *I. scapularis*, the primary Lyme disease vector in the Eastern U.S., presents an opportunity to apply the checklist as summarized in Table 2, and examine how results from extrapolation differ across multiple models. Lyme disease is the most commonly reported vector-borne disease in the U.S. [22], and infection requires that the bacteria, *Borrelia burgdorferi*, be transmitted from a competent reservoir host, such as white-footed mice (*Peromyscus leucopus*), to the tick through a blood meal, and then subsequently from the tick to a human in a later blood meal lasting more than 36 hours. Thus, tick survival and abundance are central to sustaining transmission. A number of studies have assessed the relationship of tick abundance to topography or habitat variables (e.g., slope gradients, elevation, patch size, soils, forest type), remotely-sensed data (e.g., Normalized Difference Vegetation Index or NDVI), climate/meteorological variables (e.g., temperature, day length, relative humidity), and host abundance measures (e.g., deer density, pellet counts) [23]. It is important to note that many of these models use drag sampling to estimate tick abundance, which may more accurately reflect the distribution of host-seeking ticks and thus the risk of human exposure, rather than total tick distribution.

**Figure 1:**
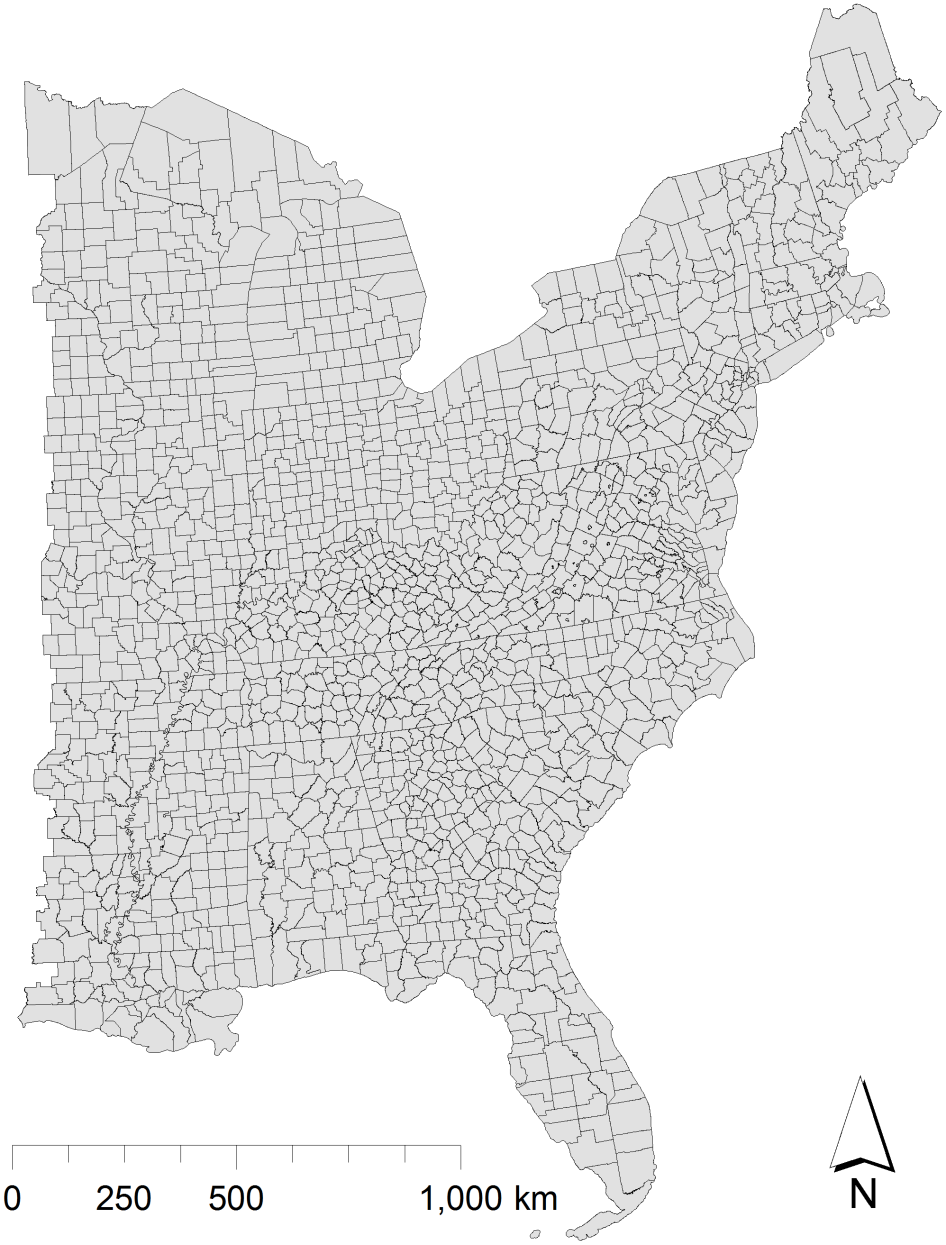
Spatial extent of Eastern United States considered in the analysis, based on 2000 U.S. Census (24.3°N to 45.9°N latitude, 93.0°W to 66.5°W longitude).

To examine issues raised by conceptual and spatial extrapolation of such models, multiple models were recreated, applied to a new domain, and their projections examined to determine the extent to which they agreed in their epidemiological and entomological risk predictions. The ability of the landscape models to predict county-level observed data was assessed, as was the extent to which agreement between models was determined by location and other geographic characteristics. Finally, the potential for improvement of model predictions through incorporation of additional information (e.g., adding variables or combining models) was examined. The analysis focused on associations between habitat variables and the county-level prevalence of either human Lyme disease or *I. scapularis*. Extrapolations were carried out on a 4×4 km grid covering the Eastern United States, starting just west of the Mississippi River (24.3°N to 45.97°N, −93.0°E to −66.88°E; Figure 1).

### Model Search and Selection

Models were selected from published research articles using habitat variables as predictors of epidemiological or entomological risk of Lyme disease in the Eastern U.S. Literature searches were carried out in PubMed using the search terms: ‘*Ixodes scapularis’* or ‘Lyme’ and ‘landscape’ or ‘habitat’ or ‘GIS’ or ‘geographic information systems’ or ‘spatial,’ and included appropriate truncation and wildcards. In addition, literature cited in Appendices 1 and 2 of the Killilea *et al.* [23] review were included. Models were then assessed according to inclusion/exclusion criteria, as follows: models must include habitat variables and *I. scapularis* or Lyme disease incidence in Eastern U.S; non-quantitative models were excluded; models that predicted survival or infection (rather than Lyme disease risk/incidence or tick presence/establishment/count) were excluded; and models that incorporated climate variables were excluded owing to the unavailability of climate data matched at the temporal and spatial resolution of the original analysis. Of approximately 30 models that examined the relationship between habitat variables and tick populations or Lyme disease in the U.S. (see Text S2), 24 were excluded on the basis of the above criteria or due to incomplete methods descriptions, dependence on data that were not available across the extrapolation area, or methods that could not be replicated due to software or processing constraints. A total of six models (heretofore termed *Tick Patch*, *Lyme Patch*, *NDVI*, *Development*, *Herbaceous*, and *Coniferous models*) were used in the analysis. The *Tick Patch model* and *NDVI model* predict tick or nymph counts per geographic unit, and the remaining four models predict odds or incidence of Lyme disease. All six models are described in Table 1 and below.

### Data Sources and Processing

Where possible, spatial data were drawn from the same year (2001) for all models. Parameter estimates for intercepts were not always provided by the literature and thus baseline counts and risks were not available. Inter-model analyses were carried out by relative pair-wise comparison of model predictions. For each model, predictor data were obtained at the same resolution as in the original analysis unless the resolution was not specified or was not available. All datasets were clipped to the extent of the full grid and projected using the Lambert Conformal Conic projection. Data processing and analyses were conducted using ArcGIS 9.3 (ESRI, Redlands, CA). Spatial join (for polygon features) and zonal statistics (for raster layers) were used to compute an average of each variable for each 4×4 km grid cell, which were then entered into the respective models (Table 1) to generate predictions in each cell. Cells located outside of U.S. boundaries (as defined by the U.S. Census Bureau) and cells comprised of greater than 50% open water (as defined by the U.S. Geological Survey) were excluded from the analysis [24], [25]. Grid predictions were also aggregated at the county level (N = 1814) to ease comparison with observed data. Detailed data sources for each model are provided below.

### Tick Patch and Lyme Patch models

National Land Cover Data (NLCD) at 30 meter resolution were obtained from the U.S. Geological Survey for the year 2001 [26]. Deciduous forest patch size and patch isolation were calculated using the program FRAGSTATS 3.3 [27]. The FragStatsBatch script [28] was used to compute class-level metrics [27] for patches of deciduous forest. All other landscape classes were set as background and ignored as specified in Brownstein *et al.* [29]. At the center of each cell in the grid, the average area of forest patches within 500 m (in hectares) and the average minimum distance between patch edges within 500 m (in meters) were calculated. The *Tick Patch model*, whose outcome is tick density, and *Lyme Patch model*, whose outcome is human Lyme disease incidence, used both patch size and patch isolation as predictors.

### NDVI model

Scaled NDVI data for June 10–25, 2001, the time period used in the original analysis [9], were obtained from the Global Land Cover Facility and were converted to true NDVI values following methods detailed elsewhere [30]. Human population data were obtained from the U.S. Census Bureau at county-level resolution for the year 2000 [24]. County population was assumed to be evenly distributed in each county. An area weighted population value, obtained from county-level population data, was applied to each grid cell, where population at a cell was estimated as the county population divided by the number of grid cells in that county. The *NDVI model* predicts number of ticks as a function of spatially averaged NDVI and human population.

### Development, Coniferous, and Herbaceous models

Soil Survey Geographic (SSURGO) data were obtained from the Natural Resources Conservation Service [31] with a variable describing each soil group's ability to support a coniferous habitat, defined as “very poor”, “poor”, “fair”, or “good.” An analogous variable for herbaceous habitat was also available. Groups described as very poor supporters of herbaceous habitats were assumed to fit into the poor-fair category described by Glass *et al.* [32]. Due to a lack of spatial orientation for soil components within each SSURGO map unit, the characteristics of the soil component which comprised the greatest proportion of the map unit were applied to the entire SSURGO map unit. Data on extent of development were obtained from the NLCD [26]. Highly developed areas were assumed to be those described as “developed, high intensity” in the NLCD. All other land cover types were assumed to be the reference category described by Glass *et al.* [32]. Of the models presented by Glass *et al.* [32], only univariate models were appropriate for inclusion in this analysis due to the presence of location-specific variables in the multivariate models. *Development*, *Coniferous*, and *Herbaceous models* predict odds of Lyme disease as a function of the extent of development, soil supporting coniferous habitat, or soil supporting herbaceous habitat, respectively.

### Observational data

Predictions from the above models were compared to county-level data on tick presence and Lyme disease risk from the U.S. Centers for Disease Control and Prevention (CDC), the definitive national dataset on Lyme disease surveillance and tick distribution in the U.S. [33], [34]. CDC categorizes tick presence for each county as none, reported (<6 ticks and 1 life stage identified), or established (≥6 ticks or >1 life stage identified), based on questionnaires sent to health officials and researchers, surveys of the MEDLINE data base, and review of National Tick Collection data. In addition, CDC categorizes Lyme disease risk as minimal/no, low, medium, or high, based on both entomologic risk obtained from tick presence and host abundance data; and risk of human exposure obtained from nationally notifiable disease surveillance.

### Statistical Analyses

Predictions were compared between models and evaluated against observational data. All comparisons are reported at the county level, although grid cell level comparisons were also conducted. County-level predictions were calculated by taking the mean of all predictions for grid cells with centroids that fell inside county boundaries, with the exception of the *NDVI model*, which predicts a tick count (in excess of the unknown baseline) rather than a risk or density and thus the sum of grid cell predictions within the county was used. State-level predictions were calculated by taking the mean of all county-level predictions within the state. Analyses were conducted using SAS 9.3 (SAS Institute Inc., Cary, NC).

### Model-model comparison

The Spearman's rank correlation coefficient (ρ) and associated p-values were calculated for each model pair to quantify the agreement between models at both county and state levels. These analyses were conducted to demonstrate how one might begin to determine the utility of extrapolated models in the absence of observational data for model validation. Assuming that no other information is available, a model-model comparison may aid in identifying outlying models that generate predictions that disagree broadly with the consensus of other models. To arrive at a value for ρ, model outputs are ranked, rankings are compared between two models (in this case, by geographic unit), and then agreement is assessed between those models over the full data set. The Spearman's rank correlation coefficient represents the level of agreement, with ρ = 1 indicating that the model outputs are in complete agreement. Spearman's rank correlation tests were performed to address dissimilarities in outcome variables that were not directly comparable in terms of units and numerical range.

The availability of hosts, the distribution of ticks across elevation gradients, the behavior of *I. scapularis*, and many other factors have been cited as sources of regional (particularly Northern vs. Southern) differences in the etiology of tick-borne human diseases in the U.S. [35]–[37]. Thus, the potential for increased model agreement in specific geographic areas was explored through analyses on subsets of the data at the county level. U.S. Census definitions were used to define these subsets: Northeast/Midwest/South, urban/rural and coastal/inland (Table 3). Elevation, categorized as high or low using the median elevation in the area of interest (calculated at the grid level), was also used to create subsets.

**Table 3:** County and state level Spearman correlation coefficients (ρ) for pair-wise model comparisons overall and for geographic sub-analyses.

Model Pair	County Level	State Level	Northeast	Midwest	South	High Elevation	Low Elevation	Coastal	Inland	Urban	Rural
**Tick Patch/Lyme Patch**	−1.00*	−1.00*	−1.00*	−1.00*	−1.00*	−1.00*	−1.00*	−1.00*	−1.00*	−1.00*	−1.00*
**Tick Patch/Development**	−0.13*	**0.36** *	−0.20*	−0.33*	−0.04	−0.34*	**0.07** *	**0.05**	−0.15*	−0.16*	−0.16*
**Tick Patch/Coniferous**	−0.13*	**0.02**	−0.26*	−0.03	−0.20*	−0.08*	−0.17*	−0.11*	−0.06*	−0.03	−0.18*
**Tick Patch/Herbaceous**	**0.22** *	**0.39** *	**0.29** *	−0.07	**0.33** *	**0.10** *	**0.32** *	**0.28** *	**0.15** *	**0.07**	**0.28** *
**Tick Patch/NDVI**	−0.14*	−0.37*	−0.14*	**0.05**	−0.19*	0.01	−0.17*	−0.10*	−0.11*	−0.08	−0.19*
**Lyme Patch/Development**	**0.14** *	−0.34	**0.22** *	**0.34** *	**0.04**	**0.35** *	−0.08*	−0.05	**0.15** *	**0.17** *	**0.16** *
**Lyme Patch/Coniferous**	**0.14** *	0.00	**0.28** *	**0.03**	**0.20** *	**0.07** *	**0.17** *	**0.12** *	**0.06** *	**0.04**	**0.18** *
**Lyme Patch/Herbaceous**	−0.23*	−0.39*	−0.32*	0.07	−0.34*	−0.10*	−0.33*	−0.29*	−0.16*	−0.09*	−0.29*
**Lyme Patch/NDVI**	**0.14** *	**0.38** *	**0.14** *	−0.05	**0.20** *	−0.01	**0.18** *	**0.10** *	**0.11** *	**0.09** *	**0.20** *
**Development/Coniferous**	**0.12** *	**0.52** *	**0.45** *	−0.05	0.10*	**0.03**	**0.23** *	**0.14** *	**0.05**	**0.17** *	**0.06**
**Development/Herbaceous**	−0.01	−0.01	−0.39*	**0.14** *	−0.01	**0.04**	−0.10*	**0.04**	**0.05**	−0.07	**0.01**
**Development/NDVI**	−0.56*	−0.21	−0.36*	−0.59*	−0.60*	−0.60*	−0.51*	−0.40*	−0.71*	−0.38*	−0.49*
**Coniferous/Herbaceous**	−0.59*	−0.48*	−0.90*	−0.45*	−0.62*	−0.54*	−0.64*	−0.71*	−0.45*	−0.67*	−0.55*
**Coniferous/NDVI**	**0.06**	**0.05**	**0.09**	**0.12** *	**0.13** *	−0.05	**0.14** *	**0.07**	−0.03	**0.04**	**0.12** *
**Herbaceous/NDVI**	−0.21*	−0.53*	−0.17*	−0.25*	−0.20*	−0.16*	−0.17*	−0.22*	−0.15*	−0.19*	−0.24*

Bolded values indicate a positive association.

*Values are significantly different from 0 (p<0.05)

### Evaluation against observations

County-level predictions for each model were compared with observational data obtained from CDC using area under the receiver operating characteristic curve (AUC) and multinomial logistic regression (MLR). AUC is a discriminatory index that is particularly useful for comparing continuous predictions to dichotomous observations because its calculation does not require subjective cut points for predictions. The statistic calculates the probability that a randomly chosen county with CDC-determined tick presence (or higher Lyme disease risk) will have a higher model-predicted score than a randomly chosen county with no CDC-determined tick presence (or lower Lyme disease risk) [38]. A model with an AUC value of 0.5 is considered to be no better than chance, while a model with an AUC value of 1 is considered to be a perfect model. Models with discriminatory power significantly better than chance were identified by an AUC p-value <0.05 in the positive direction (higher predicted values corresponding with higher observed values). Because the observational data were not dichotomous as obtained, they were categorized into “low” or “high” risk in multiple ways (see Table 4 and Table 5). To address spatial characteristics of the data, county-level predictions were regressed on CDC observed data, controlling for the effects of spatial autocorrelation with adjacent neighbors using an intrinsic conditional autoregressive model. Details of the MLR and spatial autocorrelation analyses are found in Text S2.

**Table 4:** AUC values from MLR analyses for predictive models using CDC data as gold standard.

Observational Data Set/Dichotomization	Tick Patch N = 1750	Lyme Patch N = 1750	Development N = 1814	Coniferous N = 1814	Herbaceous N = 1814	NDVI N = 1814
**Lyme disease risk**						
Minimal vs Low/Moderate/High	**0.64** *	0.65*	0.50	0.60*	0.58*	0.52
Minimal/Low vs Moderate/High	0.50	**0.51**	0.65*	0.65*	**0.49** *	**0.67** *
Minimal/Low/Moderate vs High	0.55*	**0.55** *	0.79*	0.71*	0.55*	**0.70** *
Minimal vs High	0.44	0.50	0.78*	0.75*	**0.60** *	**0.69** *
Minimal vs Moderate	**0.62** *	0.62*	0.52	0.64*	0.52	0.61*
Minimal vs Low	**0.66** *	0.67*	0.46	0.57*	**0.59** *	0.57*
Low vs High	0.64*	**0.65** *	0.80*	0.68*	0.50	**0.72** *
Low vs Moderate	0.56*	**0.57** *	0.56*	0.57*	0.57*	**0.65** *
Moderate vs High	0.59*	**0.59** *	0.77*	0.63*	**0.58** *	**0.64** *
Minimal vs Moderate/High	**0.59** *	0.59*	0.64*	0.69*	**0.55** *	**0.65** *
Minimal/Low vs High	0.54	**0.55** *	0.79*	0.71*	**0.55**	**0.71** *
Minimal vs Low/Moderate	**0.65** *	0.66*	0.47	0.58*	**0.58** *	0.55*
Low vs Moderate/High	0.60*	**0.61** *	0.67*	0.62*	0.54	**0.68** *
Minimal/Low vs Moderate	0.47	**0.48**	0.54	0.61*	0.53	**0.63** *
Low/Moderate vs High	0.64*	**0.64** *	0.80*	0.67*	**0.52**	**0.71** *
**Tick Presence**						
None vs Reported/Established	**0.60** *	0.60*	0.52	0.58*	**0.56** *	**0.52**
None/Reported vs Established	**0.54** *	0.54*	0.59*	0.64*	**0.60** *	**0.55** *
None vs Established	**0.58** *	0.58*	0.58*	0.65*	**0.61** *	**0.55** *
None vs Reported	**0.62** *	0.62*	**0.52**	0.53*	**0.53**	0.50
Reported vs Established	0.55*	**0.55** *	0.60*	0.62*	**0.58** *	**0.55** *

Bolded AUC values indicate a positive association.

*AUC values are significant (p<0.05).

**Table 5:** AUC values from MLR analyses for predictive models using CDC data as gold standard – ensemble models.

Observational Data Set/Dichotomization	Ensemble Model 1: All Models (N = 1750)	Ensemble Model 2: “Top 3” Models (N = 1750)	Ensemble Model 3: Glass et al. (1995) Models (N = 1814)
Lyme disease risk			
N vs L/M/H	0.54*	**0.61** *	0.51
N/L vs M/H	0.59*	**0.61** *	0.71*
N/L/M vs H	0.67*	**0.64** *	0.81*
N vs H	0.69*	**0.69** *	0.81
Tick presence			
A vs R/E	0.51	**0.60** *	0.53*
A/R vs E	0.56*	**0.61** *	0.59*
A vs E	0.55*	**0.63** *	0.58*

Bolded AUC values indicate a positive association.

*AUC values are significant (p<0.05).

N = none/minimal; L = low; M = moderate; H = high; A = absent/none; R = reported; E = established.

### Incorporating additional information

To test whether incorporating additional information could improve the predictive ability of models, an elevation cut-off (510 m) identified in Diuk-Wasser *et al.* [35] was incorporated into the six original models by assigning the minimum prediction value to counties above the cut-off. Three additional ensemble models were also constructed. The first included all six original models, while the second included the three models that best predicted observed data in AUC and MLR analyses. The *Coniferous*, *Herbaceous*, and *Development models* from Glass *et al.* [32] were assembled as the third ensemble model. To create ensemble statistics, predictions from each original model were ranked from lowest (1) to highest (N) and ensemble models were constructed by taking the average of the rank of each component model (thus, high ranks indicate higher valued predictions). AUC and MLR procedures were conducted using ensemble statistics as described above and the predictive ability of cut-off and ensemble models was qualitatively compared to that of the original models.

## Results

### Model-Model Comparisons

Positive, significant, though weak *ρ* were observed in six of the 15 pairwise comparisons of model prediction at the county level (p<0.01; Table 3). Two groups of models with consistent predictions emerged through these analyses. The *Tick Patch* and *Herbaceous models* were generally in agreement with each other but not with the remaining models, and vice versa. Of note, the *Tick Patch* and *Lyme Patch models* were inversely correlated (ρ = −1.0). At the state level, four of the 15 model pairs demonstrated significant evidence of agreement (p<0.05; Table 3). Grid cell level analyses showed general agreement with analyses conducted at the county level (results not shown).

Correlation sub-analyses revealed regional and topographical differences in model agreement (Table 3). While the direction of all correlations in both the Northeast and South regions remained consistent with overall results, six correlations changed direction (e.g., switched from a positive correlation to a negative correlation, or vice versa) in the Midwest. With the exception of the correlation between *Lyme Patch* and *Development*, inter-model agreement weakened at elevations above the median.

Four model pairs showed no positive correlations in either overall comparisons or any sub-analyses: *Tick Patch*/*Lyme Patch*, *Tick Patch*/*Coniferous*, *Development*/*NDVI*, and *Herbaceous*/*NDVI*. Comparisons between the *Development* and *Herbaceous models* yielded the least consistent results (the correlation coefficients for five of the nine sub-analyses were positive, while the overall correlation was negative but not significant). The most consistent correlation, that between the *Lyme Patch* and *Coniferous models*, remained positive in all sub-analyses, though the relationship was not significant in the Midwest or in urban areas.

### Evaluation Against Observations

AUC values for dichotomizations of observational data show weak agreement with modeled predictions (AUC≤0.72; Table 4). Of the 15 examined dichotomizations of CDC's Lyme disease risk data, the *NDVI model* performed significantly better than chance alone in 11 dichotomizations, while the *Lyme Patch* and *Herbaceous models* performed significantly better than chance in just under half (seven and six, respectively) of the 15 dichotomizations. In evaluations against CDC's tick presence data, the *Tick Patch* and *Herbaceous models* performed significantly better than chance in four of the five dichotomizations and the *NDVI model* in three out of five, while the *Coniferous* and *Development models* did not perform better than chance in any dichotomization of either CDC data set (Table 4). Spatial regressions showed no evidence of spatial autocorrelation across adjacent counties (results not shown).

In geographic AUC sub-analyses using four dichotomizations of CDC Lyme disease risk data, the *NDVI model* performed significantly better than chance in most geographic areas (Table S1 in Text S2). However, the *Tick Patch model* performed significantly better than chance in all Southern analyses, while the *Lyme Patch model* was the only model to demonstrate discriminatory ability in the Midwest. The *Development model* performed better than chance in only three of the 36 sub-analyses and the *Coniferous model* never performed better than chance. No best performing model emerged in geographic sub-analyses using CDC tick presence data, with multiple models demonstrating discriminatory ability in most geographic areas. The models most frequently performing better than chance were the *Tick Patch*, *Herbaceous*, and *NDVI models*. The *Lyme Patch model* again demonstrated some discriminatory ability in the Midwest, and the *Coniferous model* never performed significantly better than chance.

MLR analyses yielded similar results, with the *NDVI*, *Tick Patch*, and *Herbaceous models* producing significant positive odds ratios (ORs) against both observational data sets (Table 6 and Table S3 in Text S2). The other three models failed to demonstrate significant positive predictive ability and the *Development model* failed to converge. Sub-analyses pointed to differences in model predictive ability by geographic area, with the *NDVI* and *Herbaceous models* demonstrating significant positive predictive ability in the Northeast, and the *Lyme Patch model* demonstrating significant positive predictive ability in the Midwest (Table S2 in Text S2).

**Table 6:** Odds ratios in MLR for predictive models using CDC data as gold standard – original and ensemble models.

Outcome°	Lyme disease risk (CDC)			Tick presence (CDC)		
	OR	95% CI	AIC	OR	95% CI	AIC
**Tick Patch (N = 1750)** ∧			3761.9			3279.8
** 1v0**	**3.9** *	(2.9, 5.3)		**2.2** *	(1.6, 3)	
** 2v0**	**2.0** *	(1.2, 3.4)		**1.5** *	(1.1, 2.1)	
** 3v0**	0.9	(0.5, 1.7)				
**Lyme Patch (N = 1750)** ∧			3747.0			3274.5
** 1v0**	0.7	(0.7, 0.8)		0.8	(0.8, 0.9)	
** 2v0**	0.8	(0.7, 0.9)		0.9	(0.8, 1.0)	
** 3v0**	1.0	(0.9, 1.2)				
**Development (N = 1814)**			3927.9			3433.9
** 1v0**	0.2	(<0.001, 269.8)		15.4	(0.0, >1000)	
** 2v0**	<0.001	(<0.001, 0.2)		0.0	(0.0, 0.6)	
** 3v0**	<0.001	(<0.001, <0.001)				
**Coniferous (N = 1814)**			3915.7			3402.9
** 1v0**	0.4	(0.2, 0.6)		0.7	(0.4, 1.3)	
** 2v0**	0.2	(0.1, 0.5)		0.2	(0.1, 0.3)	
** 3v0**	0.1	(0.0, 0.1)				
**Herbaceous (N = 1814)**			3933.5			3406.6
** 1v0**	**4.8** *	(2.8, 8.2)		1.6	(0.9, 2.9)	
** 2v0**	1.4	(0.5, 3.7)		**7.0** *	(3.7, 13.2)	
** 3v0**	**4.1** *	(1.4, 11.6)				
**NDVI (N = 1814)**			3901.8			3435.8
** 1v0**	0.9	(0.9, 1.0)		1.0	(0.9, 1.1)	
** 2v0**	1.1	(1.0, 1.2)		**1.1** *	(1.0, 1.2)	
** 3v0**	**1.7** *	(1.4, 2.0)				
**Ensemble Model 1: All Models (N = 1750)**			3808.6			3293.8
** 1v0**	0.999	(0.998, 1.000)		1.000	(0.999, 1.002)	
** 2v0**	0.999	(0.997, 1.000)		0.998	(0.997, 0.999)	
** 3v0**	0.994	(0.992, 0.996)				
**Ensemble Model 2: "Top 3" Models (N = 1750)**			3776.1			3244.5
** 1v0**	**1.001** *	(1.001, 1.002)		**1.001** *	(1.001, 1.001)	
** 2v0**	**1.002** *	(1.001, 1.002)		**1.002** *	(1.001,1.002)	
** 3v0**	**1.003** *	(1.002, 1.003)				
**Ensemble Model 3: Glass et al. (1995) Models (N = 1814)**			3794.9			3417.5
** 1v0**	**1.001** *	(1.000, 1.001)		1.000	(1.000, 1.001)	
** 2v0**	0.998	(0.998, 0.999)		0.999	(0.998, 0.999)	
** 3v0**	0.994	(0.993, 0.995)				

AIC  =  Akaike information criterion; considers both model fit and complexity, used to assess goodness-of-fit.

°For Lyme Disease Risk, 0 =  minimal/no risk, 1 =  low risk/Lyme disease reported, 2 =  medium risk, 3 =  high risk. For Tick Presence, 0 =  absent/none, 1 =  reported, 2 =  established.

∧ N = 1750: Some counties had no deciduous forest; thus, patch size and patch isolation could not be calculated.

*Significant positive OR estimate: 95% CI excludes the null (1.0) and OR estimate is >1.0 (p<0.05).

### Incorporating Additional Information

Adding an elevation cut-off to predictive models increased the number of statistically significant positive AUC values and MLR ORs in most analyses (Tables S1 and S3 in Text S2). Precision was gained in MLR ORs for ensemble models that incorporated information from more than one model (Table 6). The ensemble model consisting of the three better-performing models in above analyses (*NDVI*, *Tick Patch*, and *Herbaceous*) produced all significant AUC values and MLR ORs and was positively associated with CDC data (Table 5 and Table 6). Ensemble models consisting of all six original models and the three Glass et al. [32] models produced mostly significant AUC values and MLR ORs, but were negatively associated with CDC data.

## Discussion

### Qualitative and Quantitative Assessment of Model Predictive Ability

The inter-model comparison results together with the proposed checklist for model extrapolation illustrate the value of a combined approach for identifying models suitable for extrapolation. Results from the quantitative analysis reinforced the value of the qualitative model selection checklist (Table 2), indicating that these criteria can indeed be useful for identifying the relative strengths and weaknesses of models *a priori*. For instance, based on a qualitative analysis of model selection considerations the *NDVI model* was expected to be most suitable for extrapolation to much of the studied region. The *NDVI model* presented several advantages for extrapolation over other models; these include similarity of grain size between original analysis and extrapolation, appropriate data type and categorization, and presence of the variable in the region of extrapolation. This expectation is generally borne out in comparisons to CDC observational data in both AUC (Table 4 and Table S1 in Text S2) and MLR analyses (Table 6). The *NDVI model* generated consistent positive and significant associations with both Lyme disease risk and tick presence data from CDC, henceforth jointly termed CDC-defined risk. NDVI was found in several studies to be a predictor of tick presence [39], [40], and its consistent performance in AUC comparisons to CDC data were thus anticipated. Though not uniformly significantly elevated in MLR analyses, ORs for the *NDVI model* generally increase in magnitude when moving from comparisons of low CDC-defined risk versus minimal CDC-defined risk, to comparisons of high risk versus minimal risk. This increase in OR magnitude when moving from low risk to high risk represents a monotonically increasing 'dose-response' relationship between model predictions and CDC-defined risk as estimated by the *NDVI model*. These results support the inclusion of NDVI in subsequent predictive models of tick habitat. Of note, the *NDVI model* was designed to control for human population because the detection of tick presence in this study was reliant on human hosts submitting captured ticks. The favorable performance of this model indicates that the presence and activity of the human host population, though not a traditional landscape variable, may be an important variable to consider in models of tick presence and/or Lyme disease.

In some cases, agreement of quantitative and qualitative assessments is less obvious. *Tick Patch* and *Herbaceous models* arguably perform better in MLR analysis than the *NDVI model* based solely on OR significance. However, in AUC analyses their agreement with observed data is primarily with tick presence, not Lyme disease risk. Qualitative model selection considerations indicate that univariate construction of *Coniferous*, *Herbaceous*, and *Development models* may be problematic (Table 2). In addition, the *Tick Patch* and *Lyme Patch models* were fit in Connecticut, where deciduous forest patches are numerous. However, in extrapolating these models to the remainder of the Eastern U.S., areas with few deciduous forest patches were encountered, and thus the generally uniform predictor values resulted in uniform model output and little useful information. Accordingly, the appropriateness and categorization of predictor variables were found to be lacking in these models during the preliminary, qualitative model assessment.

The *Coniferous*, *Herbaceous*, and *Development models* [32] required many assumptions in assigning values to predictors that, while effective for Baltimore County where the model was developed, may not be appropriate for other regions. For example, the poor predictive performance of the *Development model* might have been foreseen by considering the dichotomous character of the model's predictor and the quality of the original model as assessed by the qualitative criteria (Table 2). Urban areas are sparse in some areas of the U.S., resulting in a number of large rural areas with uniform predictions. Also, in the original Glass *et al.* [32] analysis, development was not a significant predictor in univariate analysis but was significant in multivariate analysis.

### Altering Models to Improve Predictive Ability

Modification of existing models through the incorporation of additional information or combining multiple models can improve the predictive ability of extrapolated models, especially for regression models that rely on just one or two predictors. Additional information may be in the form of a screening variable, such as an elevation cut-off above which no nymphs are expected to be observed [35]. In this work, the elevation cut-off at 510 meters improved agreement with observed data for most models. Combining several models into an ensemble model may also improve predictive capacity, as was demonstrated with the ensemble model comprised of the *NDVI*, *Tick Patch*, and *Herbaceous models*, termed the *“top 3” ensemble model* (Table 5 and Table 6). The failure of other ensemble models to demonstrate improved predictive capacity highlights the efficacy of using qualitative (Table 2), in addition to quantitative (e.g., Table 4), criteria to inform selection of models for the ensemble.

### Inter-model Comparison: The Effect of Spatial Extent and Scale

Model-model correlations highlight the regional nature of the models studied. In regional sub-analyses, model-model correlations in the South were generally weak, and models that agreed in the majority of sub-analyses often disagreed in the Midwest. These findings point to the challenges in extrapolating models developed in a single region, as all models in the present study were developed and fit in Canada and the Northeast U.S. (Table 1). Importantly, several studies have shown that the number of reported cases of Lyme disease is lower in the Southeast than in the Northeast [22]. Differences in *I. scapularis* abundance, host composition, tick behavior, and other factors may explain the lower number of Lyme disease cases in the South [36], [40]. Additional studies of the relationship between landscape variables and both tick abundance and Lyme disease occurrence in the South, following the guidelines presented here, would aid model extrapolators in better characterizing Lyme disease in the Eastern U.S.

Though the *Tick Patch model* showed high overall agreement with observed data in all analyses, a comparison to the closely related *Lyme Patch model* from the same project reveals some interesting discrepancies that suggest non-stationarity in space and time. Brownstein *et al.* 's [29] *Tick Patch* and *Lyme Patch models* were inversely correlated (*ρ* = −1.0), and the authors acknowledged that this suggests a lack of a positive association between the density of tick populations and the incidence of Lyme disease. However, Lyme disease risk and tick presence have been shown to be correlated elsewhere [41], and were positively correlated in the CDC observational data sets presented here. While *Tick Patch* and *Lyme Patch models* were fit to regional data, they were not validated with reserved data in the same or different regions or time periods [29]. Taken in concert with our findings, this highlights problems associated with non-stationarity when extrapolating models developed in a single region and time period [42]. Modelers ideally consider all relevant variables and obtain data representing the full range of each variable in the production of niche or habitat models, yet data limitations are common and resulting models may have limited applicability outside the spatial and temporal range in which they were fit.

## Conclusions

Previous work has shown that factors such as scale, data quality, and modeling technique are important to consider when extrapolating ecological models. Such qualitative considerations may have value in predicting the quantitative suitability of models applied to new questions or locations, especially where researchers have time or budget constraints and elect to apply information from previously published work. Investigators who are interested in extrapolating a model but are unable to carry out a comprehensive quantitative comparison of all candidate models can use the qualitative considerations detailed here to identify the most promising models for extrapolation (e.g., Table 2). Further refinement of models selected using these criteria may be achieved by developing an ensemble model or applying further literature-based selection criteria. Such systematic consideration of these criteria by both producers and consumers of ecological models will facilitate model development and usefulness, while strengthening collaboration between these two groups.

## Supporting Information

Text S1 (PDF)

Text S2 (PDF)
