# Supplementary material for: Inter-Model Comparison of the Landscape Determinants of Vector-Borne Disease: Implications for Epidemiological and Entomological Risk Modeling
Source: PLoS One. 2014 Jul 29;9(7):e103163. doi: 10.1371/journal.pone.0103163 (PMC4114569; doi:10.1371/journal.pone.0103163)
Supplement: Text S1 — (PDF) [file pone.0103163.s001.pdf]

TEXT S1 FOR

Inter-model comparison of the landscape determinants of vector-borne disease: implications for epidemiological and entomological risk modeling

Alyson Lorenz[1], Radhika Dhingra[1], Howard H. Chang[2], Donal Bisanzio[3], Yang Liu[1], Justin V. Remais[1,4*]

[1] Department of Environmental Health, Rollins School of Public Health, Emory University, Atlanta, GA, USA.

[2] Department of Biostatistics and Bioinformatics, Rollins School of Public Health, Emory University, Atlanta, GA, USA.

[3] Department of Environmental Sciences, Emory University, Atlanta, GA, USA.

[4] Program in Population Biology, Ecology and Evolution, Graduate Division of Biological and Biomedical Sciences, Emory University, Atlanta, GA, USA.

[*]Email: justin.remais@emory.edu

Development of a checklist for the evaluation of models for extrapolation

Checklists have been successfully used in a variety of disciplines in order to improve the quality and reliability of work. In both public health and ecology, checklists have been used to organize a broad literature (e.g., Bosshard 2000; de Groot *et al.* 2002; de Groot *et al.* 2003), to assess the quality of epidemiologic studies, (e.g., Downs and Black 1998) or ecological management strategies (e.g., Lindenmayer *et al.* 2008), and to facilitate communication and comparison of related work (e.g., Bosshard 2000; Vandenbroucke *et al.* 2007). To improve the methodological quality of landscape model extrapolation in vector-borne disease risk assessment, we provide checklist criteria for the qualitative assessment of models being considered for extrapolation. Here, we briefly describe model characteristics that appear on the checklist, and distinguish between conceptual extrapolation and spatial extrapolation.

Conceptual extrapolation

Applying existing models to new research questions—a conceptual extrapolation—raises the importance of key characteristics of the original analysis, including its predictor and outcome variables, scale and resolution, reproducibility, and data quality and availability.

Characteristics of the original analysis. Ascertaining the quality of the original analysis includes assessment of the modeling technique used, the species being modeled,

and the model selection technique employed. For species distribution modeling, multi-model comparisons have found that different modeling techniques (e.g., generalized linear models, artificial neural networks, etc.) exhibit varying generalizability, robustness and predictive capacity (Pearson *et al.* 2006; Randin *et al.* 2006; Dormann *et al.* 2008), and thus no one class of model is universally preferred for conceptual extrapolation (Dormann *et al.* 2008; Jeschke and Strayer 2008; Elith and Leathwick 2009). Models drawn from research conducted on the exact species of interest are preferred (MacArthur 1958; Elith and Leathwick 2009); however, at times, a model describing a related organism may be useful (Raxworthy *et al.* 2003). The model selection technique employed in the original analysis should be examined, as models selected using fit as the sole criterion may not be appropriate given that over-fit models are often uninformative beyond the spatial or temporal confines of the original analysis (Ginzburg and Jensen 2004; Hitchcock and Sober 2004). Additional criteria such as parsimony, agreement with previous findings or theories, and predictive capability can strengthen the model selection process (Ginzburg and Jensen 2004).

Predictor and outcome variables. The predictor and outcome variables that appear in a model, and how they were selected and treated in the analysis, are primary considerations for determining the model's value and generalizability. Using indirect predictor variables (e.g., elevation), which act as proxies for other influential variables

(e.g., temperature, humidity), may decrease predictive ability (Austin and Smith 1989; Elith and Leathwick 2009). Proxy variables, often chosen because of limited data availability (Dormann *et al.* 2008), should be selected only after a careful assessment of the appropriateness of the proxy in light of the underlying variables of interest. The data type (continuous, categorical, nominal, ordinal, etc.) utilized in the original analysis may affect the utility of a candidate model, as inaccurate assumptions about the true distribution the data (Sauerbrei *et al.* 2007), and inappropriate categorical transformations (Brooker *et al.* 2002; Araujo *et al.* 2005; Royston *et al.* 2006), can contribute to bias yielding a model unsuitable for conceptual extrapolation.

Scale and resolution. At different spatial and temporal scales and resolutions of analysis, both the magnitude and direction of a relationship between predictor and outcome variables can vary (Chase and Leibold 2002; Jackson *et al.* 2006). In the spatial domain, such variation may be produced by disappearance of non-dominant habitat types with decreasing resolution, which influences variable collinearity, or bias associated with alternate methods of averaging over areas (Turner *et al.* 1989; Benson and MacKenzie 1995). Models which were developed using data at coarse resolution may have low predictive ability if used at fine scale (Kunin 1998), for instance. Thus, a model fit at a scale that is mismatched with the scale of the new research question may be inappropriate for conceptual extrapolation. Likewise, the predictive ability of species

distribution models across timescales may also be limited if, for instance, species rapidly evolve leading to changes in habitat suitability and violating the assumption of niche conservatism (Pearson and Dawson 2003; Chaves and Koenraadt 2010).

Reproducibility. Logistical issues in reproducing candidate models also arise. When researchers fail to specify the full model, or do not completely define the methods used for data transformation or for dealing with missing data, it may be impossible to re-apply the model unless the original authors can be consulted. Such unspecified models were excluded from our analyses. Access to appropriate computer applications and versions may also be a barrier to successful replication of the model.

Data quality and availability. Finally, the quality of the data used to fit candidate models is an important consideration when choosing a model for conceptual extrapolation (Dormann *et al.* 2008). In many cases, the most robust parameter estimates result from species distribution models fit to datasets that balance quantity with quality, i.e., achieving a sufficient number of points with adequate quality (Dormann *et al.* 2008).

Spatial extrapolation

Extrapolation of models across spatial domains beyond what was included in the original analysis requires many of the same considerations as when undertaking conceptual extrapolation, but must also take into account additional issues related to

the spatial aspects of model variables, choice of predictor and outcome variables, and spatial extent, as described briefly here.

Spatial aspects of model variables. Issues surrounding spatial extrapolation of species distribution models are covered in a wide body of literature that has been comprehensively reviewed elsewhere (e.g., Miller *et al.* 2004; Peters *et al.* 2004). Several key issues routinely arise, such as the fact that suitable habitats tend to be more varied in the center of the geographical range occupied by a species, leading the relationships between predictors and outcomes to differ by range position (Peterson *et al.* 2000; Swihart *et al.* 2003; Randin *et al.* 2006). Thus applying a model fit at the center of a species' range to an area at the edge of the range could lead to overestimation of presence at the edge, for instance, or underestimation of presence at the center. Models must therefore incorporate locations throughout a species' range (e.g. Webber *et al.* 2011), and testing models against independent observations rather than observations used in model fitting is of course preferred (Fielding and Bell 1997; Guisan and Zimmerman 2000).

Choice of predictor and outcome variables. The relevance, range, and relationships between predictor and outcome variables often differ across space. Issues of spatial stationarity of predictor-outcome relationships are well-described elsewhere (e.g., Brunsdon *et al.* 1998). Predictor variables may become irrelevant in new geographical

areas where these variables are missing or where their explanatory power decreases (Rodder and Lotters 2010). The use of indirect or proxy predictors, which may fail to describe true habitat preferences of a species, may also limit a model's spatial transferability (Austin and Smith 1989; Austin 2002). Even when the exact predictor of interest can be measured across the extrapolation zone, its numerical range may fall outside the range over which the original model was fit, limiting model performance (Peters *et al.* 2004). Finally, the categorization of outcome variables may have limited meaning in new locations. For example, the category of 'high' risk for mosquito bites may represent a different range of risk values in Albany, New York than in New Orleans, Louisiana.

Spatial extent. The spatial extent of the data used to fit the model, and that of the new domain, raise similar issues. Research conducted over a large area in which there is significant variance in predictors and outcomes may lead to a more robust and transferable model, particularly with respect to extreme values (Wiens 1989). Additionally, predictor data must be available across the extrapolation zone, and attention must be paid to missing data and data collected at a resolution that differs from the intended resolution of the analysis (Kistemann *et al.* 2002).

References

Araujo MB, Whittaker RJ, Ladle RJ*, et al.* 2005. Reducing uncertainty in projections of extinction risk from climate change. Global Ecology and Biogeography **14**: 529-538.

Austin MP. 2002. Spatial prediction of species distribution: an interface between ecological theory and statistical modelling. Ecological Modelling **157**: 101-118.

Austin MP, and Smith TM. 1989. A new model for the continuum concept. Vegetatio **83**: 35-47.

Benson BJ, and MacKenzie MD. 1995. Effects of sensor spatial resolution on landscape structure parameters. Landscape Ecology **10**(2): 113-120.

Bosshard A. 2000. A methodology and terminology of sustainability assessment and its perspectives for rural planning. Agriculture Ecosystems & Environment **77**(1-2): 29-41.

Brooker S, Hay SI, and Bundy DA. 2002. Tools from ecology: useful for evaluating infection risk models? Trends Parasitol **18**(2): 70-74.

Brunsdon C, Fotheringham S, and Charlton M. 1998. Geographically Weighted Regression. Journal of the Royal Statistical Society: Series D (The Statistician) **47**(3): 431-443.

Chase JM, and Leibold MA. 2002. Spatial scale dictates the productivity-biodiversity relationship. Nature **416**(6879): 427-430.

Chaves LF, and Koenraadt CJ. 2010. Climate change and highland malaria: fresh air for a hot debate. Q Rev Biol **85**(1): 27-55.

de Groot RS, Wilson MA, and Boumans RMJ. 2002. A typology for the classification, description and valuation of ecosystem functions, goods and services. Ecological Economics **41**(3): 393-408.

de Groot V, Beckerman H, Lankhorst GJ*, et al.* 2003. How to measure comorbidity: a critical review of available methods. Journal of Clinical Epidemiology **56**(3): 221-229.

Dormann CF, Purschke O, Garcia Marquez JR*, et al.* 2008. Components of uncertainty in species distribution analysis: a case study of the Great Grey Shrike. Ecology **89**(12): 3371-3386.

Downs SH, and Black N. 1998. The feasibility of creating a checklist for the assessment of the methodological quality both of randomised and non-randomised studies of health care interventions. Journal of Epidemiology and Community Health **52**(6): 377-384.

Elith J, and Leathwick JR. 2009. Species distribution models: ecological explanation and prediction across space and time. Annu Rev Ecol Evol Syst **40**: 677-697.

Fielding AH, and Bell JF. 1997. A review of methods for the assessment of prediction errors in conservation presence/absence models. Environmental Conservation **24**(1): 38-49.

Ginzburg LR, and Jensen CX. 2004. Rules of thumb for judging ecological theories. Trends Ecol Evol **19**(3): 121-126.

Guisan A, and Zimmerman NE. 2000. Predictive habitat distribution models in ecology. Ecological Modelling **135**: 147-186.

Hitchcock C, and Sober E. 2004. Prediction versus accommodation and the risk of overfitting. British Journal for the Philosophy of Science **55**: 1-34.

Jackson LE, Levine JF, and Hilborn ED. 2006. A comparison of analysis units for associating Lyme disease with forest-edge habitat. Community Ecology **7**(2): 189-197.

Jeschke JM, and Strayer DL. 2008. Usefulness of bioclimatic models for studying climate change and invasive species. Ann N Y Acad Sci **1134**: 1-24.

Kistemann T, Dangendorf F, and Schweikart J. 2002. New perspectives on the use of Geographical Information Systems (GIS) in environmental health sciences. Int J Hyg Environ Health **205**(3): 169-181.

Kunin WE. 1998. Extrapolating Species Abundance Across Spatial Scales. Science **281**(5382): 1513-1515.

Lindenmayer D, Hobbs RJ, Montague-Drake R*, et al.* 2008. A checklist for ecological management of landscapes for conservation. Ecol Lett **11**(1): 78-91.

MacArthur RH. 1958. Population ecology of some warblers of northeastern coniferous forests. Ecology **39**(4): 599-619.

Miller JR, Turner MG, Smithwick EAH*, et al.* 2004. Spatial extrapolation: the science of predicting ecological patterns and processes. BioScience **54**(4): 310-320.

Pearson RG, and Dawson TP. 2003. Predicting the impacts of climate change on the distribution of species: are bioclimate envelope models useful? Global Ecology and Biogeography **12**: 361-371.

Pearson RG, Thuiller W, Araujo MB*, et al.* 2006. Model-based uncertainty in species range prediction. Journal of Biogeography **33**: 1704-1711.

Peters DPC, Herrick JE, Urban DL*, et al.* 2004. Strategies for ecological extrapolation. OIKOS **106**(3): 627-636.

Peterson AT, Egbert SL, Sanchez-Cordero V*, et al.* 2000. Geographic analysis of conservation priority: endemic birds and mammals in Veracruz, Mexico. Biological Conservation **93**: 85-94.

Randin CF, Dirnbock T, Dullinger S*, et al.* 2006. Are niche-based species distribution models transferable in space? Journal of Biogeography **33**: 1689-1703.

Raxworthy CJ, Martinez-Meyer E, Horning N*, et al.* 2003. Predicting distributions of known and unknown reptile species in Madagascar. Nature **426**(6968): 837-841.

Rodder D, and Lotters S. 2010. Explanative power of variables used in species distribution modelling: an issue of general model transferability or niche shift in the invasive Greenhouse frog (*Eleutherodactylus planirostris*). Naturwissenschaften **97**(9): 781-796.

Royston P, Altman DG, and Sauerbrei W. 2006. Dichotomizing continuous predictors in multiple regression: a bad idea. Stat Med **25**(1): 127-141.

Sauerbrei W, Royston P, and Binder H. 2007. Selection of important variables and determination of functional form for continuous predictors in multivariable model building. Stat Med **26**(30): 5512-5528.

Swihart RK, Gehring TM, and Kolozsvary MB. 2003. Responses of 'resistant' vertebrates to habitat loss and fragmentation: the importance of niche breadth and range boundaries. Diversity and Distributions **9**: 1-18.

Turner MG, O'Neill RV, Gardner RH*, et al.* 1989. Effects of changing spatial scale on the analysis of landscape pattern. Landscape Ecology **3**: 153-162.

Vandenbroucke JP, von Elm E, Altman DG*, et al.* 2007. Strengthening the Reporting of Observational Studies in Epidemiology (STROBE) Explanation and Elaboration. Epidemiology **18**(6): 805-835.

Webber BL, Yates CJ, Le Maitre DC*, et al.* 2011. Modelling horses for novel climate courses: insights from projecting potential distributions of native and alien Australian acacias with correlative and mechanistic models. Diversity and Distributions **17**(5): 978-1000.

Wiens JA. 1989. Spatial scaling in ecology. Functional Ecology **3**(4): 385-397.
