# Supplementary material for: Inter-Model Comparison of the Landscape Determinants of Vector-Borne Disease: Implications for Epidemiological and Entomological Risk Modeling
Source: PLoS One. 2014 Jul 29;9(7):e103163. doi: 10.1371/journal.pone.0103163 (PMC4114569; doi:10.1371/journal.pone.0103163)
Supplement: Text S2 — (PDF) [file pone.0103163.s002.pdf]

TEXT S2 FOR

Inter-model comparison of the landscape determinants of vector-borne disease: implications for epidemiological and entomological risk modeling

Alyson Lorenz[1], Radhika Dhingra[1], Howard H. Chang[2], Donal Bisanzio[3], Yang Liu[1], Justin V. Remais[1,4*]

[1] Department of Environmental Health, Rollins School of Public Health, Emory University, Atlanta, GA, USA.

[2] Department of Biostatistics and Bioinformatics, Rollins School of Public Health, Emory University, Atlanta, GA, USA.

[3] Department of Environmental Sciences, Emory University, Atlanta, GA, USA.

[4] Program in Population Biology, Ecology and Evolution, Graduate Division of Biological and Biomedical Sciences, Emory University, Atlanta, GA, USA.

[*]Email: justin.remais@emory.edu

Section 1: Details of Multinomial Logistic Regression

Critics of using area under the receiver operating characteristic curve (AUC) for model evaluation stress that the method does not consider the fit of the model and recommend complementing AUC analyses with other procedures (Lobo *et al.* 2007). Multinomial logistic regression (MLR) was used to address other aspects of predictive ability including model fit. MLR accommodates a categorical outcome with multiple (e.g., the observational CDC data) levels without forcing ordinality (Ananth and Kleinbaum 1997; Kleinbaum *et al.* 2010). The observational CDC data contain four categories of Lyme disease risk and three categories of tick presence. The MLR model for tick presence (Y), and a single predictor variable (X), for instance, can be expressed as:

$$\ln\left[\frac{P(Y=1\,|\,\mathrm{X})}{P(Y=0\,|\,\mathrm{X})}\right]=\alpha_1+\beta_1 X$$

$$\ln\left[\frac{P(Y=2\,|\,\mathrm{X})}{P(Y=0\,|\,\mathrm{X})}\right]=\alpha_2+\beta_2 X$$

$$\ln\left[\frac{P(Y=3\,|\,\mathrm{X})}{P(Y=0\,|\,\mathrm{X})}\right]=\alpha_3+\beta_3 X$$

MLR was used to generate the odds ratio (OR) for a particular outcome category (e.g., established tick presence as categorized by CDC) compared to a reference outcome category (e.g., absence of ticks as categorized by CDC), given particular predictor variables (e.g., the *NDVI model* or the *Lyme Patch model*). ORs and 95% confidence intervals (CIs) were calculated for each outcome level compared to the reference (no tick

presence or no/minimal Lyme disease risk). Akaike information criterion (AIC), which considers both model fit and complexity, was computed and used to assess goodness-of-fit.

Results of the MLR analyses

ORs and 95% CIs from MLR models comparing model predictions to CDC observations across the Eastern United States are presented in Table 6 of the main text and Table S3 of the this Text S2. The *Tick Patch*, *Herbaceous*, and *NDVI models* all yielded at least one OR that was significantly greater than one in comparisons to both observed Lyme disease risk and observed tick presence data. The other three models failed to demonstrate significant positive predictive ability and the *Development model* failed to converge. Based on AIC in MLR analyses of Lyme disease risk and tick presence, *Tick Patch* and the *"Top 3"* ensemble model had the best fit among the individual models and ensemble models, respectively, that were positively associated with observed data (Table 6 of the main text). In comparison to observed Lyme disease risk, *Tick Patch* AIC showed better fit than the *"Top 3"* ensemble model whereas the opposite was true for observed tick presence. When moving from low to high levels of CDC-defined Lyme disease risk or tick presence, ORs for the *NDVI model* increased incrementally in magnitude, while ORs for the *Tick Patch model* decreased incrementally in magnitude.

ORs and CIs from MLR for selected geographic sub-analyses are presented in Table S2 (not all data shown). The only sub-analysis that produced all significantly positive ORs for both Lyme disease risk and tick presence was the *Tick Patch model* in the South. The *Herbaceous model* produced at least one significantly positive OR in all sub-analyses except for the Midwest and high elevation areas, while the *NDVI model* achieved the same in all sub-analyses except for the Midwest, South, and rural areas. The *Lyme Patch model* was the only model with significant positive ORs in the Midwest. OR estimates for the *Development model* were unstable across all sub-analyses, though some estimates were significantly positive in rural areas. The *Coniferous model* did not produce any significant positive ORs.

Section 2: Details of Spatial Autocorrelation Analyses

For spatial analyses, the reported classifications were grouped into all possible dichotomizations (e.g. for Lyme disease, dichotomizations included minimal no vs. low, medium and high; minimal/no and low vs. medium and high; and minimal/no, low, and medium vs. high). As above, model outcome was spatially averaged at the county level.

A spatial logistic model was also used to fit CDC observed data to model outcome for n counties, according to the following model:

$$logit[\, P(y_i = 1)] = \alpha + \lambda_i + \beta X_i,$$

where y_i is the dichotomized observed Lyme disease category; α is the overall baseline risk; λ_i is the county-specific spatial random effects; and β represents log odds ratio associated with measures of population response (X_i). We modeled the spatial random effects to control for potential spatial confounders using an intrinsic conditional autoregressive (CAR) model (Lee 2011). Let $i \sim i'$ denote that county i and county i' are spatial neighbors sharing a common boundary.

The CAR model is often formulated by the conditional distribution of λ_i given its neighbors. Let m_i denote the number of neighbors of county i, the conditional distribution is Gaussian with mean $\frac{1}{m_i}\sum_{i\sim i'}\lambda_{i'}$ and variance τ^2/m_i. Therefore the CAR model assumes each λ_i is a spatial average of its neighbors and parameter τ^2 controls the degree of spatial similarly. To ensure identifiablility, we impose the constraint $\Sigma\lambda_i = 0$.

To demonstrate sensitivity to spatial autocorrelation, the fit models were compared to the analogous generalized linear model (GLM), fit according to the following model:

$$logit[\,P(y_i = 1)] = \alpha + \beta X_i$$

Results of the spatial autocorrelation analyses

Among GLM models with significant (α=0.05) parameter estimates (β), the inclusion of spatial autocorrelation resulted in slight deviation in the parameter estimates (-2.0% to 3.7%) compared to that resulting from the GLM. Those parameters whose estimates

show the greatest degree of dissonance ($\leq \pm 10\%$) did not have significant p-values (α=0.05) in the GLM model. All models produced small estimates of τ^2 (0.003, 0.034) as compared to the parameter estimate or the model's intercept, which indicates that the contribution of spatial correlation to the model outcome's ability to predict CDC-observed data is negligible.

Section 1 and 2 References

Ananth CV, and Kleinbaum DG. 1997. Regression models for ordinal responses: a review of methods and applications. Int J Epidemiol **26**(6): 1323-1333.

Kleinbaum DG, Klein M, and Rihl Pryor E. 2010. Logistic Regression: A Self-Learning Text. New York, Springer.

Lee D. 2011. A comparison of conditional autoregressive models used in Bayesian disease mapping. Spatial and Spatio-temporal Epidemiology **2**: 78-89.

Lobo JM, Jimenez-Valverde A, and Real R. 2007. AUC: a misleading measure of the performance of predictive distribution models. Global Ecology and Biogeography: 1-7.

Section 3: Literature Review of Lyme Models

The following are a listing of source papers for all models subjected to the inclusion/exclusion criteria and considered for the analyses carried out in this study.

1. Allan BF, F Keesing, RS Ostfeld: **Effect of forest fragmentation on Lyme disease risk**. *Conservation Biology* 2003 **17**(1): 267-272.
2. Amerasinghe FP, NL Breisch, AF Azad, WF Gimpel, M Greco, K Neidhardt, B Pagac, J Piesman, J Sandt, TW Scott, K Sweeney: **Distribution, Density, and Lyme Disease Spirochete Infection in Ixodes dammini (Acari: Ixodidae) on White-Tailed Deer in Maryland**. Journal of Medical Entomology 1992 **29**(1): 54-61.
3. Brownstein JS, DK Skelly, TR Holford, D Fish: **Forest fragmentation predicts local scale heterogeneity of Lyme disease risk**. *Oecologia* 2005 **146**: 469-175.
4. Bunnell JE, SD Price, A Das, TM Shields, GE Glass: **Geographic Information Systems and spatial analysis of adult *Ixodes scapularis* (Acari: Ixodidae) in the Middle Atlantic region of the U.S.A.** *Journal of Medical Entomology* 2003 **40**(4): 570-576.
5. Cromley EK, ML Cartter, RD Mrozinski, SH Ertel: **Residential setting as a risk factor for Lyme disease in a hyperendemic region**. *American Journal of Epidemiology* 1998 **147**(5): 472-477.
6. Duffy DC, DD Clark, SR Campbell, S Gurney, R Perello, N Simon: **Landscape patterns of abundance of *Ixodes scapularis* (Acari: Ixodidae) on Shelter Island, New York**. *Journal of Medical Entomology* 1994 **31**(6): 875-879.
7. Frank DH, D Fish, FH Moy: **Landscape features associated with Lyme disease risk in a suburban residential environment**. *Landscape Ecology* 1998 **13**: 27-36.
8. Ginsberg HW, E Zhioua, S Mitra, J Fischer, PA Buckley, F Verret, HB Underwood, FG Buckley: **Woodland type and spatial distribution of nymphal *Ixodes scapularis* (Acari: Ixodidae)**. *Population Ecology* 2004 **33**(5): 1266-1273.
9. Glass GE, BS Schwartz, JM Morgan, DT Johnson, PM Noy, E Israel: **Environmental risk factors for Lyme disease identified with**

Geographic Information Systems. *American Journal of Public Health* 1995 **85**(7): 944-948.

10. Glass GE, FP Amerasinghe, JM Morgan, TW Scott: **Predicting *Ixodes scapularis* abundance on white-tailed deer using Geographic Information Systems**. *Am. J. Trop. Med. Hyg.* 1994 **51**(5): 538-544.
11. Guerra M, E Walker, C Jones, S Paskewitz, MR Cortinas, A Stancil, L Beck, M Bobo, U Kitron: **Predicting the risk of Lyme disease: Habitat suitability for *Ixodes scapularis* in the north central United States**. *Emerging Infectious Diseases* 2002 **8**(3): 289-297.
12. Horobik V, F Keesing, RS Ostfeld: **Abundance and *Borrelia burgdorferi*-infection prevalence of nymphal *Ixodes scapularis* ticks along forest-field edges**. *EcoHealth* 2007 **3**: 262-268.
13. Jackson LE, ED Hilborn, JC Thomas. **Towards landscape design guidelines for reducing Lyme disease risk**. *International Journal of Epidemiology* 2006 **35**: 315-322.
14. Jackson LE, JF Levin, ED Hilborn: **A comparison of analysis units for associating Lyme disease with forest-edge habitat**. *Community Ecology* 2006 **7**(2): 189-197.
15. Jordan RA and RL Schulze: **Deer browsing and the distribution of *Ixodes scapularis* (Acari: Ixodidae) in central New Jersey forests**. *Environmental Entomology* 2005 **34**(4): 801-806.
16. Kitron U, JJ Kazmierczak: **Spatial analysis of the distribution of Lyme disease in Wisconsin**. *American Journal of Epidemiology* 1997 **145**(6): 558-566.
17. Kitron U, JK Bouseman, CJ Jones: **Use of the ARC/INFO GIS to study the distribution of Lyme disease ticks in an Illinois county**. *Preventive Veterinary Medicine* 1991 **11**: 243-248.
18. Lubelczyk CB, SP Elias, PW Rand, MS Holman, EH Lacombe, RP Smith: **Habitat associations of *Ixodes scapularis* (Acari: Ixodidae) in Maine**. *Community and Ecosystem Ecology* 2004 **33**(4): 900-906.
19. Mannelli A, U Kitron, CJ Jones, TL Slajchert: **Influence of season and habitat on *Ixodes scapularis* infestation on white-footed mice in northwestern Illinois**. *The Journal of Parasitology* 1994 **80**(6): 1038-1042.
20. Ogden NH, L Trudel, H Artsob, IK Barker, G Beauchamp, DF Charron, et al. ***Ixodes scapularis* ticks collected by passive surveillance in Canada: Analysis of geographic distribution and infection with Lyme Borreliosis**

agent *Borrelia burgdorferi*. *Journal of Medical Entomology* 2006 **43**(3): 600-609.

21. Ostfeld RS, OM Cepeda, KR Hazler, MC Miller: **Ecology of Lyme disease: Habitat associations of ticks (*Ixodes scapularis*) in a rural landscape**. *Ecological Applications* 1995 **5**(2): 353-361.
22. Raizman EA, JD Holland, LM Keefe, MH Moro: **Forest and surface water as predictors of *Borrelia burgdorferi* and its vector *Ixodes scapularis* (Acari: Ixodidae) in Indiana**. *Journal of Medical Entomology* 2010 **47**(3): 458-465.
23. Schulze TL, RA Jordan, RW Hung: **Comparison of *Ixodes scapularis* (Acari: Ixodidae) populations and their habitats in established and emerging Lyme disease areas in New Jersey**. *Journal of Medical Entomology* 1998 **35**(1): 64-70.
24. Stafford KC and LA Magnarelli: **Spatial and temporal patterns of *Ixodes scapularis* (Acari: Ixodidae) in Southeastern Connecticut**. *Journal of Medical Entomology* 1993 **30**(4): 762-771.

Table S1 - AUC values for predictive models using CDC data (Lyme disease risk and Tick presence) as gold standard, before and after applying elevation cut-off and sub-analyses

Area	Observational Data Set / Dichotomization	Tick Patch	Lyme Patch	Development	Coniferous	Herbaceous	NDVI
Overall without Elevation Cut-Off (N=1814)							
	Lyme disease risk						
	N vs L/M/H	0.64*†	0.65*†	0.50	0.60*	0.58*	0.52
	N/L vs M/H	0.50†	0.51†	0.65*	0.65*	0.49	0.67*
	N/L/M vs H	0.55*†	0.55*†	0.79*	0.71*	0.55*	0.70*
	N vs H	0.44†	0.50†	0.78*	0.75*	0.60*	0.69*
	Tick presence						
	A vs R/E	0.60*†	0.60*†	0.52	0.58*	0.56*	0.52
	A/R vs E	0.54*†	0.54*†	0.59*	0.64*	0.60*	0.55*
	A vs E	0.58*†	0.58*†	0.58*	0.65*	0.61*	0.55*
Overall with Elevation Cut-Off (N=1814)							
	Lyme disease risk						
	N vs L/M/H	0.65*	0.65*	0.56*	0.44	0.62*	0.53*
	N/L vs M/H	0.50	0.51	0.37	0.64*	0.53	0.66*
	N/L/M vs H	0.54	0.54*	0.25	0.68*	0.57*	0.71*
	N vs H	0.57*	0.57*	0.30	0.69*	0.64*	0.72*
	Tick presence						
	A vs R/E	0.60*	0.61*	0.52	0.56*	0.60*	0.55*
	A/R vs E	0.55*	0.55*	0.45	0.61*	0.62*	0.57*
	A vs E	0.58*	0.58*	0.47	0.61*	0.64*	0.58*
Northeast (N=217)							
	Lyme disease risk						
	N vs L/M/H	0.56	0.56	0.72*	0.58	0.58	0.63
	N/L vs M/H	0.51	0.52	0.67*	0.61	0.63*	0.62*
	N/L/M vs H	0.50	0.51	0.85*	0.66*	0.65*	0.67*
	N vs H	0.57	0.57	0.94*	0.65	0.64	0.72*
	Tick presence						
	A vs R/E	0.56	0.57	0.30	0.57	0.59	0.69*
	A/R vs E	0.52	0.53	0.73*	0.65*	0.66*	0.62*
	A vs E	0.56	0.57	0.77*	0.62*	0.64*	0.70*
Midwest (N=544)							
	Lyme disease risk						
	N vs L/M/H	0.55*	0.55*	0.47	0.50	0.57*	0.53
	N/L vs M/H	0.49	0.50	0.54	0.55	0.58*	0.50
	N/L/M vs H	0.67*	0.68*	0.60	0.61	0.60*	0.60
	N vs H	0.68*	0.68*	0.61	0.60	0.63*	0.58
	Tick presence						

A vs R/E	0.52	**0.52**	**0.50**	**0.50**	0.56*	0.50
A/R vs E	0.61*	**0.61***	**0.60***	**0.51**	0.55	**0.48**
A vs E	0.60*	**0.60***	**0.59***	**0.51**	0.56	**0.48**
South (N=1053)						
Lyme disease risk						
N vs L/M/H	**0.78***	0.79*	**0.54***	0.60*	**0.66***	0.60*
N/L vs M/H	**0.64***	0.63*	0.75*	0.66*	**0.53**	**0.59**
N/L/M vs H	**0.66***	0.65*	0.78*	0.64*	0.52	**0.61**
N vs H	**0.83**	0.83*	0.77*	0.70*	**0.58**	**0.58**
Tick presence						
A vs R/E	**0.71***	0.72*	**0.55***	0.57*	**0.63***	0.58*
A/R vs E	**0.69***	0.70*	**0.47**	0.63*	**0.67***	0.55
A vs E	**0.74***	0.74*	**0.49**	0.64*	**0.69***	0.57*
Urban (N=619)						
Lyme disease risk						
N vs L/M/H	**0.59***	0.59*	0.58*	0.66*	**0.62***	**0.52**
N/L vs M/H	0.50	**0.51**	0.65*	0.66*	**0.54**	**0.65***
N/L/M vs H	0.54	**0.55**	0.73*	0.71*	**0.58***	**0.66***
N vs H	0.47	**0.47**	0.74*	0.77*	**0.65***	**0.66***
Tick presence						
A vs R/E	**0.60***	0.60*	0.56*	0.67*	**0.61***	**0.56***
A/R vs E	**0.55**	0.54	0.58*	0.70*	**0.62***	**0.57***
A vs E	**0.58***	0.58*	0.59*	0.72*	**0.64***	**0.58***
Rural (N=1195)						
Lyme disease risk						
N vs L/M/H	**0.67***	0.68*	**0.57***	0.56*	**0.56***	0.57*
N/L vs M/H	0.50	**0.51**	**0.46**	0.63*	0.52	**0.63***
N/L/M vs H	0.59	**0.60***	**0.45**	0.63*	0.52	**0.63***
N vs H	0.48	**0.48**	**0.49**	0.66*	**0.52**	**0.60**
Tick presence						
A vs R/E	**0.60***	0.60*	**0.55***	0.53	**0.54***	0.53
A/R vs E	**0.54**	0.54	**0.54**	0.57*	**0.59***	0.54
A vs E	**0.57***	0.58*	**0.56***	0.57*	**0.59***	0.55
Coastal (N=538)						
Lyme disease risk						
N vs L/M/H	**0.55**	0.55	0.56*	0.63*	**0.57***	**0.56***
N/L vs M/H	0.66*	**0.67***	0.72*	0.60*	0.56*	**0.69***
N/L/M vs H	0.68*	**0.69***	0.82*	0.60*	0.58*	**0.75***
N vs H	0.61*	**0.62***	0.83*	0.68*	0.52*	**0.75***
Tick presence						
A vs R/E	**0.53**	0.53	0.55*	0.62*	**0.57***	**0.57***
A/R vs E	0.54	**0.54**	0.63*	0.62*	**0.56***	**0.61***
A vs E	0.51	**0.51**	0.62*	0.64*	**0.58***	**0.61***

**Inland (N=1276)**						
Lyme disease risk						
N vs L/M/H	**0.65***	0.66*	**0.55***	0.54*	**0.55***	0.53
N/L vs M/H	**0.54**	0.53	**0.48**	0.61*	0.57*	**0.68***
N/L/M vs H	0.56	**0.57**	**0.47**	0.62*	0.55	**0.67***
N vs H	0.46	**0.47**	**0.49**	0.63*	0.53	**0.66***
Tick presence						
A vs R/E	**0.60***	0.61*	**0.53**	**0.48**	**0.51**	**0.52**
A/R vs E	0.47	0.53	**0.53**	0.54	**0.52**	**0.55**
A vs E	**0.57***	0.57*	**0.54**	0.54	**0.52**	**0.55**
**High Elevation (N=956)**						
Lyme disease risk						
N vs L/M/H	**0.60***	0.60*	**0.51**	0.57*	0.49	**0.54***
N/L vs M/H	**0.56***	0.56*	**0.52**	0.62*	0.51	**0.63***
N/L/M vs H	0.55	**0.56**	**0.45**	0.65*	0.48	**0.69***
N vs H	0.50	**0.50**	**0.46**	0.66*	0.48	**0.70***
Tick presence						
A vs R/E	**0.59***	0.59*	**0.49**	0.57*	0.49	**0.59***
A/R vs E	0.49	**0.49**	**0.53**	0.59*	**0.52**	**0.57***
A vs E	0.46	**0.46**	**0.52**	0.60*	**0.52**	**0.58***
**Low Elevation (N=858)**						
Lyme disease risk						
N vs L/M/H	**0.64***	0.64*	0.54	0.59*	**0.58***	**0.52**
N/L vs M/H	0.57*	**0.58***	0.85*	0.72*	**0.57***	**0.67***
N/L/M vs H	0.61*	**0.62***	0.90*	0.70*	**0.52**	**0.72***
N vs H	0.48	**0.49**	0.91*	0.75*	**0.58***	**0.72***
Tick presence						
A vs R/E	**0.55***	0.55*	0.54	0.56*	**0.55***	**0.54***
A/R vs E	**0.52**	0.52	0.65*	0.64*	**0.61***	**0.57***
A vs E	**0.54**	0.54	0.64*	0.64*	**0.61***	**0.57***

* AUC values are significant ($p<0.05$)

† N=1750, some counties had no deciduous forest so patch isolation could not be calculated

Bolded AUC values indicate a positive association

N=none/minimal; L=low; M=moderate; H=high; A=absent/none; R=reported; E=established

Table S2 - Odds ratios in MLR for predictive models using CDC data as gold standard – selected sub-analyses

Area	Northeast (N=217)				Midwest (N=544)			
Outcome°	Lyme disease risk (CDC)		Tick presence (CDC)		Lyme disease risk (CDC)		Tick presence (CDC)	
	OR	95% CI	OR	95% CI	OR	95% CI	OR	95% CI
Tick Patchˆ								
1v0	1.8	(0.1, 41.2)	2.0	(0.3, 14.7)	0.4	(0.3, 0.7)	0.9	(0.5, 1.6)
2v0	2.5	(0.1, 53.1)	1.9	(0.3, 11.8)	0.8	(0.4, 1.8)	0.3	(0.1, 0.6)
3v0	1.7	(0.1, 36.9)			0.1	(0.0, 0.6)		
Lyme Patchˆ								
1v0	0.9	(0.4, 1.8)	0.8	(0.5, 1.3)	1.2*	(1.1, 1.4)	1.0	(0.9, 1.1)
2v0	0.8	(0.4, 1.6)	0.8	(0.6, 1.2)	1.0	(0.9, 1.2)	1.4*	(1.1, 1.6)
3v0	0.8	(0.4, 1.7)			1.7*	(1.1, 2.6)		
Development								
1v0	<0.001	(<0.001, >1000)	21.2	(0.0, >1000)	0.0	(<0.001, >1000)	0.3	(<0.001, >1000)
2v0	<0.001	(<0.001, >1000)	0.7	(0.0, 45.4)	-1.7	(<0.001, >1000)	>1000	(<0.001, >1000)
3v0	<0.001	(<0.001, >1000)			>1000	(<0.001, >1000)		
Coniferous								
1v0	1.5	(0.1, 41.3)	4.6	(0.6, 37)	1.1	(0.4, 3.3)	1.5	(0.4, 5.0)
2v0	0.7	(0.0, 17.6)	0.3	(0.0, 1.4)	1.0	(0.2, 5.6)	1.2	(0.2, 5.7)
3v0	0.1	(0.0, 1.8)			0.4	(0.0, 6.8)		
Herbaceous								
1v0	0.3	(0.0, 10.5)	0.2	(0.0, 2.7)	0.3	(0.1, 0.9)	0.3	(0.1, 0.9)
2v0	1.1	(0.0, 37.4)	9.1*	(1.1, 75.2)	0.2	(0.0, 1.1)	0.4	(0.1, 1.8)
3v0	9.2	(0.3, 306)			0.0	(0.0, 1.3)		
NDVI								
1v0	1.1	(0.9, 1.4)	1.3*	(1.0, 1.5)	1.0	(0.9, 1.1)	1.0	(0.9, 1.1)
2v0	1.2	(0.9, 1.5)	1.2*	(1.0, 1.4)	1.0	(0.9, 1.1)	1.0	(0.9, 1.1)
3v0	1.3*	(1.0, 1.7)			1.3	(0.9, 1.8)		

* Significant positive OR estimate: 95% CI excludes the null (1.0) and OR estimate is >1.0 (p<0.05).

ˆ N=217 in Northeast and N=543 in Midwest, some counties had no deciduous forest so patch size and patch isolation could not be calculated.

° For Lyme Disease Risk, 0 = minimal/no risk, 1 = low risk/Lyme disease reported, 2 = medium risk, 3 = high risk. For Tick Presence, 0 = absent/none, 1 = reported, 2 = established.

Table S3 - Odds ratios in MLR for predictive models using CDC data as gold standard, before and after applying elevation cut-off

Area		Overall (N=1814)				With Elevation Cut-Off Applied (N=1814)			
Outcome°		Lyme disease risk (CDC)		Tick presence (CDC)		Lyme disease risk (CDC)		Tick presence (CDC)	
		OR	95% CI	OR	95% CI	OR	95% CI	OR	95% CI
Tick Patch^									
	1v0	3.9*	(2.9, 5.3)	2.2*	(1.6, 3.0)	4.3*	(3.2, 5.7)	2.3*	(1.8, 3.1)
	2v0	2.0*	(1.2, 3.4)	1.5*	(1.1, 2.1)	2.1*	(1.3, 3.3)	1.8*	(1.3, 2.4)
	3v0	0.9	(0.5, 1.7)			1.4*	(0.8, 2.3)		
Lyme Patch^									
	1v0	0.7	(0.7, 0.8)	0.8*	(0.8, 0.9)	0.7	(0.7, 0.8)	0.8	(0.8, 0.9)
	2v0	0.8	(0.7, 0.9)	0.9	(0.8, 1.0)	0.8	(0.8, 0.9)	0.9	(0.8, 0.9)
	3v0	1.0	(0.9, 1.2)			0.9	(0.8, 1.1)		
Development									
	1v0	0.2	(<0.001, 269.8)	15.4	(0.0, >1000)	10.8*	(5.2, 22.4)	4.2*	(2.2, 8.1)
	2v0	<0.001*	(<0.001, 0.2)	0.0*	(0.0, 0.6)	1.8*	(1.0, 3.4)	4.2*	(2.0, 8.8)
	3v0	<0.001*	(<0.001, <0.001)			2.8*	(1.2, 6.6)		
Coniferous									
	1v0	0.4	(0.2, 0.6)	0.7	(0.4, 1.3)	1.5*	(1.0, 2.2)	1.6*	(1.0, 2.6)
	2v0	0.2	(0.1, 0.5)	0.2	(0.1, 0.3)	0.7	(0.4, 1.3)	0.6	(0.4, 0.9)
	3v0	0.1	(0.0, 0.1)			0.3	(0.2, 0.7)		
Herbaceous									
	1v0	4.8*	(2.8, 8.2)	1.6	(0.9, 2.9)	9.2*	(5.4, 15.7)	2.6*	(1.4, 4.7)
	2v0	1.4	(0.5, 3.7)	7.0*	(3.7, 13.2)	2.7*	(1.0, 7.1)	10.5*	(5.6, 19.7)
	3v0	4.1*	(1.4, 11.6)			8.0*	(2.9, 22.3)		
NDVI									
	1v0	0.9	(0.9, 1.0)	1.0	(0.9, 1.1)	1.1*	(1.0, 1.1)	1.1*	(1.0, 1.1)
	2v0	1.1	(1.0, 1.2)	1.1*	(1.0, 1.2)	1.1*	(1.0, 1.2)	1.2*	(1.1, 1.2)
	3v0	1.7*	(1.4, 2.0)			1.7*	(1.4, 2.0)		

^ N=1750, some counties had no deciduous forest so patch size patch isolation could not be calculated

* Significant positive OR estimate: 95% CI excludes the null (1.0) and OR estimate is >1.0 (p<0.05)

° For Lyme Disease Risk, 0 = minimal/no risk, 1 = low risk/Lyme disease reported, 2 = medium risk, 3 = high risk. For Tick Presence, 0 = absent/none, 1 = reported, 2 = established.
